# Supplementary material for: Mycobacterium Transcriptional Factor BlaI Regulates Cell Division and Growth and Potentiates β-Lactam Antibiotic Efficacy Against Mycobacteria
Source: Microorganisms. 2025 Sep 25;13(10):2245. doi: 10.3390/microorganisms13102245 (PMC12565901; doi:10.3390/microorganisms13102245)
Supplement: Supplementary file 1 [file microorganisms-13-02245-s001.zip › microorganisms-3749036-supplementary.pdf]

Supplementary Figure

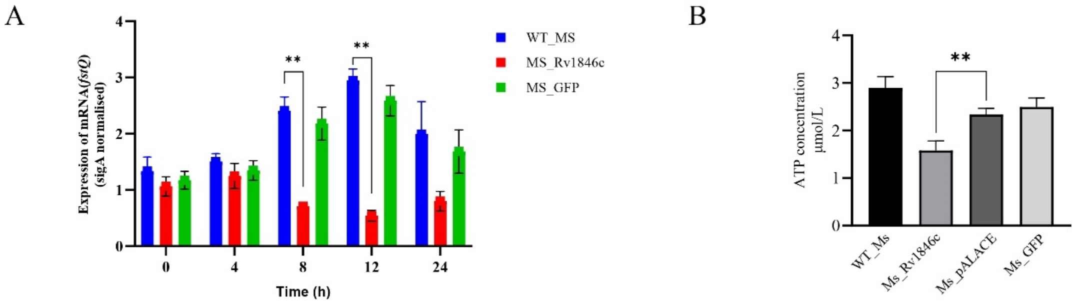

Supplementary Fig.1. (A) The relative expression levels of ftsQ in the BlaI overexpression strain (Ms_Rv1846c) and the blank control (Ms_GFP) were detected by qPCR (n=3, mean ± standard deviation). (B) The bar graph shows the ATP content (in relative fluorescence units, RFU) of Ms_Rv1846c, Ms_GFP strain and wild-type (WT) after induction for 8 hours (n=3, mean ± standard deviation).

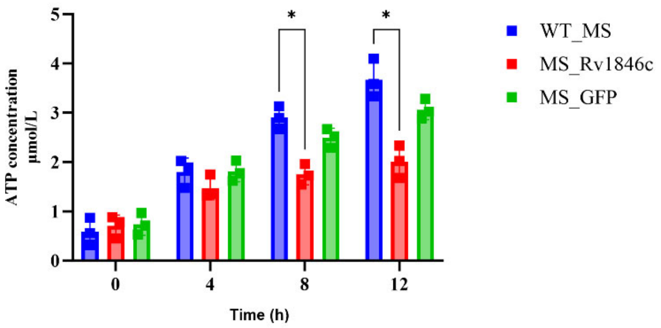

Supplementary Fig.2. Time-course analysis of intracellular ATP levels and cell length in BlaI-overexpressing and control M. smegmatis strains. Intracellular ATP levels (relative fluorescence units, RFU) in Ms_Rv1846c (BlaI-overexpressing) and WT_MS (control) strains measured at 0, 4, 8, and 12 hours post-acetamide induction. ATP levels were quantified using an ATP Assay Kit (Beyotime, S0026) and normalized to total protein concentration.

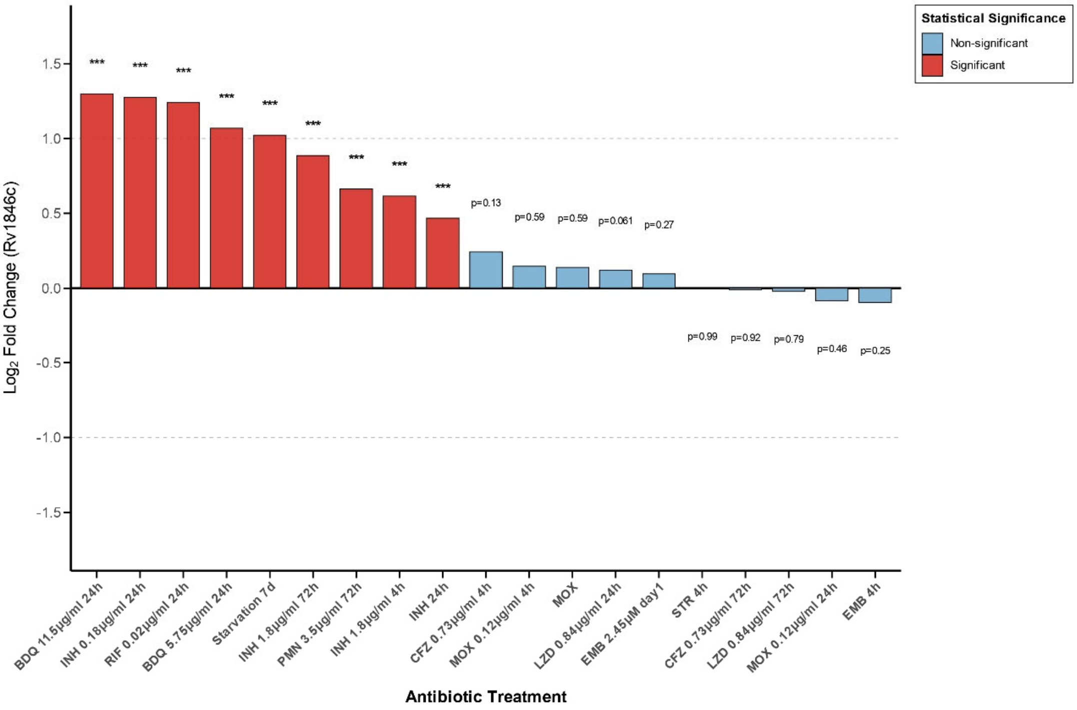

Supplementary Fig.3. Differential Expression of Rv1846c Under Stress Conditions Linked to ATP Metabolism and Cell Division.

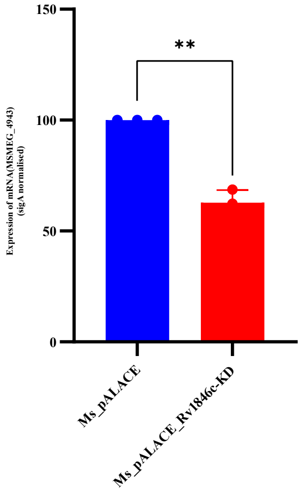

Supplementary Fig.4. Quantitative real-time PCR (qRT-PCR) analysis of MSMEG_4943 transcription levels in the CRISPRi-mediated knockdown strain (Ms_pALACE_Rv1846c-KD) and the empty plasmid control strain (Ms_pALACE).

Table S1

Primer	Upstream primer (5'➞3')	Downstream primer (5'➞3')
ftsZ	GCGGTGAAGATCCTGAAGA	TCAGGCGATCTTGTCGTTGT
ftsQ	ACGACCTGCTGCTGAAGAT	TCGATGCGCTTGATGTTCT
ftsB	GCTGCTGCTGAAGAAGGTG	CGGATGATGATGATGATGGT
ftsL	GAAGAAGAAGCGGAAGAAGG	CTTGCTGCTGCTGCTGTTGT
sepF	GCGGAAGAAGAAGAAGAAGG	CTTGCTGCTGCTGCTGTTGT
ftsI	GAAGAAGAAGCGGAAGAAGG	CTTGCTGCTGCTGCTGTTGT
ftsK	GCTGCTGCTGAAGAAGGTG	CGGATGATGATGATGATGGT
ftsE	ACGACCTGCTGCTGAAGAT	TCGATGCGCTTGATGTTCT
wag31	GCGGTGAAGATCCTGAAGA	TCAGGCGATCTTGTCGTTGT
ftsH	GAAGAAGAAGCGGAAGAAGG	CTTGCTGCTGCTGCTGTTGT
murC	GCTGCTGCTGAAGAAGGTG	CGGATGATGATGATGATGGT
murD	ACGACCTGCTGCTGAAGAT	TCGATGCGCTTGATGTTCT
murE	GCGGTGAAGATCCTGAAGA	TCAGGCGATCTTGTCGTTGT
murF	GAAGAAGAAGCGGAAGAAGG	CTTGCTGCTGCTGCTGTTGT
murG	GCTGCTGCTGAAGAAGGTG	CGGATGATGATGATGATGGT
ripA	ACGACCTGCTGCTGAAGAT	TCGATGCGCTTGATGTTCT
clwM	GCGGTGAAGATCCTGAAGA	TCAGGCGATCTTGTCGTTGT
chiZ	GAAGAAGAAGCGGAAGAAGG	CTTGCTGCTGCTGCTGTTGT
pknA	GCTGCTGCTGAAGAAGGTG	CGGATGATGATGATGATGGT
pknB	ACGACCTGCTGCTGAAGAT	TCGATGCGCTTGATGTTCT
